# Prevention and screening during the COVID-19 pandemic: qualitative findings from the BETTER WISE project

**DOI:** 10.1186/s12875-022-01954-x

**Published:** 2023-01-23

**Authors:** N. Sopcak, M. Wong, C. Fernandes, D. Ofosu, I. Khalil, D. Manca

**Affiliations:** 1 Department of Family Medicine, University of Alberta, Edmonton, Canada; 2 MAP Centre for Urban Health Solutions, St. Michael’s Hospital, Toronto, Canada

**Keywords:** COVID-19, Primary prevention, Chronic disease, Primary care, Patient care team, Qualitative research

## Abstract

**Background:**

The COVID-19 pandemic challenged healthcare systems worldwide and disrupted primary care, particularly prevention, screening, and lifestyle counselling. BETTER WISE is a comprehensive and structured approach that proactively addresses cancer and chronic disease prevention and screening (CCDPS), including cancer survivorship and screening for poverty and lifestyle risks for patients aged 40 to 65. Patients from 13 primary care clinics (urban, rural, and remote) in Alberta, Ontario, and Newfoundland & Labrador, Canada were invited for a 1-hour visit with a prevention practitioner (PP), a member of the primary care team with specialized training in CCDPS to provide patients an overview of eligible screening and assist with lifestyle counselling. This qualitative sub-study describes how the COVID-19 pandemic impacted BETTER WISE in a constantly changing medical landscape.

**Methods:**

We conducted 17 focus groups and 48 key informant interviews with a total of 132 primary care providers (PPs, physicians, allied health professionals, and clinic staff) over three different time points to better understand their perspectives on the BETTER WISE project. We also received 585 patient feedback forms of the 1005 patients who agreed to participate in the study. We also collected field notes and memos and employed thematic analysis using a constant comparative method focused on the impact of the pandemic on BETTER WISE.

**Results:**

We identified four themes related to how the COVID-19 pandemic impacted the BETTER WISE study: 1) Switch of in-person visits to visits over the phone; 2) Lack of access to preventive care and delays of screening tests; 3) Changes in primary care providers’ availability and priorities; 4) Mental health impacts of the pandemic on patients and primary care providers.

**Conclusions:**

The COVID-19 pandemic had and, at the time of writing, continues to have an impact on primary care, particularly on prevention, screening, and lifestyle counselling. Despite structural, procedural, and personal challenges throughout different waves of the pandemic, the primary care clinics participating in BETTER WISE were able to complete the study. Our results underscore the importance of the role of primary care providers in adapting to changing circumstances and support of patients in these challenging times.

**Trial registration:**

This qualitative study is a sub-component of the BETTER WISE pragmatic, cRCT, trial registration ISRCTN21333761 (date of registration 19/12/2016).

## Background

When the World Health Organization (WHO) declared a pandemic due to the outbreak of the coronavirus disease of 2019 (COVID-19) in March 2020, strained health care systems faced increased demand due to testing, vaccinations, and increased patient admissions to hospitals and intensive care units worldwide [1]. Since then, primary care providers have been navigating a constantly shifting landscape with often changing regulations and demands [2]. Since primary care is characterized by first point of contact, comprehensiveness, continuity, and coordination with other health care providers, primary care providers have a prominent role in the COVID-19 pandemic in Canada [3]. However, emerging research suggests that primary prevention and screening has been interrupted and delayed by the COVID-19 pandemic worldwide. For instance, a study conducted in Spain found that less diagnoses of cancers and cardiovascular risk factors were a result of patients having less face-to-face contacts with their family doctors who had to prioritize COVID-19 care [4]. Similarly, postponement of surgical procedures seemed to have led to adverse patient outcomes [5] and due to the disruption of colorectal cancer screening, deaths are projected to increase in Canada, Australia, and the Netherlands [6]. DeJonge et al. [6] suggest that “disruption to screening programmes will have a substantial effect on the absolute number of colorectal cancer deaths between 2020 and 2050”.

This qualitative study is a sub-component of the BETTER WISE pragmatic, cluster randomized controlled trial (cRCT) and describes how the COVID-19 pandemic impacted the BETTER WISE (Building on Existing Tools to Improve Cancer and Chronic Disease Prevention and Screening in Primary Care for Wellness of Cancer Survivors and Patients) project, a Canadian-based cRCT with a qualitative evaluation and economic assessment [7]. The BETTER WISE intervention is a comprehensive and structured approach that proactively addresses cancer and chronic disease prevention and screening (CCDPS), including cancer survivorship and screening for poverty and lifestyle risks for patients 40 to 65 years of age. BETTER WISE builds on previous BETTER studies, which have shown improved chronic disease prevention and screening outcomes and that patients appreciate this personalized approach [8–11].

In BETTER WISE, 1005 patients from 13 primary care clinics in Alberta, Ontario, and Newfoundland & Labrador, Canada were invited for a one-hour, one-on-one, in-person visit with a prevention practitioner (PP), a member of the primary care team (e.g., registered nurse, licensed practical nurse, dietitian). PPs received training in the BETTER WISE approach and the BETTER WISE tools [10, 12], and met with patients for a collaborative conversation to discuss the patients’ risk of cancer, diabetes and heart disease, including associated lifestyle factors such as diet, physical activity, smoking and alcohol, and screening for poverty. Survivors of breast, colorectal, and/or prostate cancer also received individualized counselling on cancer surveillance [7]. Patients aged 40 to 65 with and without cancer history were randomized into receiving either a prevention visit with a PP or standard care (i.e. wait-list control). Patients in the control group were offered a visit with a PP 1 year after their enrollment in the study. The primary outcome was measured at 12-months using a composite index [7]. The BETTER WISE study was at midpoint of implementation when the COVID-19 pandemic started in March of 2020 and due to provincial restrictions, most clinics were closed to in-person patient appointments and had to shift to phone or virtual visits. This shift in protocol included BETTER WISE visits as well, but since the study was already offering telephone visits, the study could carry on.

Our preliminary results suggest that prior to the start of the pandemic the intervention group showed significant improvement in comparison to the control group, as measured by a composite index of completed actions. This effect, however, was not only minimized with the onset of the pandemic, but in fact reversed at the tail end of the study (Manca et al., manuscript submitted for publication). In the following, we describe how the COVID-19 pandemic impacted BETTER WISE from the perspective of primary care providers (PPs, physicians, allied health professionals, and clinic staff) and patients who participated in the study.

## Methods

### Study setting

Due to our involvement in the BETTER WISE pragmatic cRCT we had a pre-established relationship with 13 primary care clinics (urban, rural, and remote) in Alberta, Ontario, and Newfoundland & Labrador, Canada and their PPs. Our PPs consisted of four licensed or registered practical nurses, three registered nurses, one pharmacist, one kinesiologist, one clinical medical assistant, one clinic coordinator, one nurse practitioner, and one registered dietician. All PPs were trained by the BETTER WISE team in delivering the intervention using the BETTER WISE approach and toolkit and in how to communicate with patients in a neutral and non-judgmental way when addressing patients’ health status and lifestyle [7]. The PPs were also trained and certified in Brief Action Planning, a structured and practical approach “to help people make action plans to address the aspects of their health or situation that are most important to them” [13].

Prior to a patient’s prevention visit, PPs reviewed patients’ medical charts and answers to their self-administered health survey, which included questions on health status, family and medical history, social determinants of health, and lifestyle behaviours, such as smoking, physical activity, and diet. When the patients came in for their one-hour prevention visit, the PPs then provided patients with a comprehensive snapshot of their health, eligible screening, and assisted patients with setting S.M.A.R.T. (specific, measurable, attainable, realistic, time-based) lifestyle or screening-related goals, helping to guide patients towards healthy lifestyle modifications or further resources as needed (e.g., referral to a social worker, a dietician, a smoking cessation program, or back to the family physician, etc.) [7].

### Participants and recruitment

Physicians and clinic staff, including the PPs, at each of the 13 participating primary care clinics, were invited to participate in focus groups and key informant interviews to share their perspectives on BETTER WISE at three different time points of the study: at baseline when PPs had recruited their patients and just started implementing BETTER WISE (mid to late 2018), at follow up mid-way through the study (mid to late 2020), and at the end of the study (early to mid 2021), when PPs had completed all of their prevention visits with patients. Baseline focus groups were conducted exclusively in-person. The focus groups that took place at mid-point and at the end of the study were all conducted online over Zoom®. One-on-one key informant interviews were also conducted at each time point, over the telephone or Zoom®, depending on the participant’s preference, and were scheduled alongside with focus groups based on individuals’ availability. Physicians, PPs, and other staff were invited to participate in a focus group at all time points to understand their perspectives as the study progressed. PPs were also invited to participate in one-on-one key informant interviews at baseline, follow-up, and end of study to enable them to share their perspectives over time. Other key informants were invited for interviews depending on their availability. Focus group and interviews ranged from 20 to 90 minutes with an average time of 45–60 minutes. We obtained written informed consent from all participants either in-person or via e-mail, and we used a semi-structured interview guide to ask about each primary care setting’s context, processes, and how the implementation (including facilitators and barriers) of BETTER WISE impacted patients and providers.

Patients were invited to provide anonymous feedback using a short feedback form that they received following each of their prevention visits. Patients received an information letter along with the feedback form, which informed them that by completing the feedback form and submitting it to the team they were providing implied consent to participate in the qualitative component of the study.

### Data collection

The baseline focus groups conducted in-person allowed participants to meet the BETTER WISE team and become acquainted with the components of the BETTER WISE study. They also allowed the study team to capture group thinking and to get an overall sense of the respective context of each clinic setting. The follow up with online focus groups and key informant interviews provided opportunities for more in-depth conversations. Focus group size ranged from two (mini-focus group) to 19 participants and were conducted by the first author and qualitative research lead (NS) and the provincial research coordinator who observed group dynamics and took notes. All key informant interviews were conducted by NS only. We used a semi-structured interview guide to capture clinic contexts, processes, and the impact of the BETTER WISE implementation. All focus groups and interviews were audio recorded and transcribed with the use of the software Trint®. Both the qualitative research lead (NS) and a research assistant (DO) proofread and edited all transcripts. Field notes from the focus groups (collected by the qualitative research lead and the provincial coordinators) and memos from different team members (documenting thoughts about the data and the research process) were also collected and used in the analysis process. Patients also had the opportunity to fill out a feedback form after their prevention visit that included demographic information, patients’ expectations, what patients liked about the visit, what they would have liked to be different, and a general comment box. Patients also received an information letter along the feedback form that indicated that feedback was voluntary, anonymous, and that filling out the form implied giving consent to providing feedback. A research assistant (DO) and a research coordinator (MW) entered all feedback form responses into REDCap®, an electronic data base and a generated Excel Microsoft® spreadsheet for data analysis.

### Data analysis

We conducted a thematic sub-analysis by filtering out all the data related to the COVID-19 pandemic using Microsoft® Word to organize and code the transcripts and memos. The qualitative research lead (NS) did a first round of coding by sorting all the data related to the COVID-19 pandemic from the interviews and focus groups, and three investigators (MW, DO, and IK) identified preliminary codes and themes from the data in a second round of coding. DO filtered the patient responses related to the COVID-19 pandemic from REDCap® and compiled them in a generated Excel spreadsheet for analysis. Lastly, MW combined all data sets and conceptualized the data into major themes in a third round of coding, and NS refined the themes into four major themes in a last round of coding.

The term data saturation refers to the point in data analysis where researchers find similar information identified in participants’ responses, where no new information is found and enough data has been collected to generate a theme. We received a large number of patient feedback forms returned (*n* = 585) and had a large number of individuals participate in our focus groups and key informant interviews (*n* = 132). We determined that data saturation was reached as we focused our analysis on the themes that were most salient and emerged from all three different data sets.

### Rigor of study methods

Involving physicians, a variety of members of primary care teams, PPs, and patients contributed to the rigor of this qualitative study, as we could triangulate different data points and compare different perspectives. Using different data collection strategies (focus groups, key informant interviews, patient feedback forms), and diverse settings (urban, rural, remote) in different provinces (Alberta, Ontario, Newfoundland & Labrador) also provided richness and diversity to the data and enhanced our analysis. Our team consisted of clinicians and researchers with previous experience with qualitative research. All authors met for analysis meetings and discussed the data until consensus on the four themes was reached.

## Results

We conducted 17 focus groups with 128 individuals from the 13 participating primary care settings (16% male, 84% female; 54% from Alberta [AB], 30% from Ontario [ON], and 16% from Newfoundland & Labrador [NL]). 48 key informant interviews were conducted by telephone with 25 healthcare providers, including two men and 23 women over three different data collection points (baseline, follow up, end of study interview). The key informants included 16 PPs, seven physicians, one clinic director, and one research assistant. 12 participants were from AB, seven from ON, and six from NL. See Table 1 for focus group and key informant interview participant characteristics.

**Table 1:** Selected characteristics of study participants involved in 17 focus groups and 48 key informant interviews

**Total # of participants (focus groups)**	***N* = 128**
Characteristic	No. (%)
Gender	
Male	20 (16%)
Female	108 (84%)
Profession	
Primary Care Physician	42 (33%)
Admin/MOA/clerical staff	29 (23%)
Registered Nurse	16 (12%)
Clinic manager / coordinator / director	14 (11%)
Other clinicians (social worker, pharmacist, dieticians)	10 (8%)
Licensed Practical Nurse / Registered Practical Nurse	9 (7%)
Nurse Practitioner / Physician Assistant	6 (5%)
Family Medicine Residents	2 (1%)
Province	
Alberta	69 (54%)
Ontario	38 (30%)
Newfoundland & Labrador	21 (16%)
**Total # of participants (key informant interviews) (2 PPs completed a second interview when they left role)**	***N* = 25**
Characteristic	
Gender	
Male	2
Female	23
Profession	
Registered Nurse	3
Licensed or Registered Practical Nurse	7
Physician	7
Nurse practitioner	1
Dietician	1
Pharmacist	1
Clinic director	1
Clinic coordinator	1
Clinic medical assistant	1
Kinesiologist	1
Research Assistant	1
Province	
Alberta	12
Ontario	7
Newfoundland & Labrador	6
Note: We had a total of 132 participants. 128 participants were part of focus groups, 25 participants were part of key informant interviews, most of them participated in both.	

Overall, 1005 patients agreed to a BETTER WISE prevention visit, which comprised 63% of eligible patients contacted. We received 585 patient feedback forms over three data collection points (36% male, 64% female; 62% from AB, 19% from ON, 19% from NL). We identified four key themes in how the COVID-19 pandemic impacted the BETTER WISE study: 1) Switch of in-person visits to visits over the phone; 2) Lack of access to preventive care and delays of screening tests; 3) Changes in primary care providers’ availability and priorities; 4) Mental health impacts of the pandemic on patients and primary care providers.

### Theme 1: switch of in-person visits to visits over the phone

The first and most immediate change to the BETTER WISE study was that prevention visits with a PP could no longer be in-person. The prevention visits for the intervention group were allocated 60 minutes at baseline, 12-months, and 24-months and focused on reviewing the results from patients’ completed BETTER WISE surveys, patients’ charts, and on identifying eligible screening tests and setting lifestyle goals if patients wished to do so. Follow-up visits at 6-month and 18-month time points were typically 15–30 minutes and focused mainly on checking in on existing goals and flagging outstanding screening tests. Control group patients received one prevention visit after patients in the intervention group had received their 12-month visit. Prior to the pandemic, best practice was that annual prevention visits (i.e. baseline, 12-months, and 24-months) be done in-person, while follow-up visits (i.e., 6-month and 18-month) could be done in-person or over the phone. In March 2020, access to clinics was limited and clinics had to shift to telephone visits, which included BETTER WISE visits. Since BETTER WISE had previously offered telephone visits for follow up appointments to patients in the intervention group, the change was not overly disruptive, and the study could continue. However, some of the control group patients had not yet had the opportunity of an in-person visit. Furthermore, while some clinic sites had previously offered phone visits, they were at the discretion of the patient and the PP. Some clinics had their majority of visits in-person and PPs commented on the difficulties of having to switch to phone visits, including issues such as not being able to reach people, poor cell phone reception in rural areas, and the perception that it was more difficult to have a personal connection and to read people’s body language.

*“I don’t think they’re as effective, over the phone. (…) Because it’s not easy talking to people about stuff that stresses them out, over the phone. [I]t’s way more effective in person. Because you can read their body language. (…) Plus, I get this feeling, sometimes, that they’re not even paying attention. You know what I mean; that they’re distracted.”*

*[PP, KI020, NL].*

*“I think it was hard really trying to understand and communicate without having to see the person. Because one patient can say something, and they can have very different body language and they can mean it in a different way. Another patient might be totally different. So, it took a lot of time for me to adjust. Because I’d be confused about, you know, is this a goal that they feel confident about? Even if I ask them a question about how likely they think they are to be consistent with the goal? Some patients are so different, and you can tell sometimes by body language that they might be unsure. But over the phone you can’t see that. So, I’d have to ask a lot of additional questions to try to figure that out. That was a learning experience.”*

*[PP, KI046, AB].*

With the change to phone visits, PPs were no longer able to take measurements for the study such as weight, waist circumference, and blood pressure. Although PPs asked patients to take at-home measurements as an alternative where patients had access to equipment, it affected our ability to collect these measurements and the accuracy of the data collected.

*“I feel that when we’re in-person we have to get the weight check, we can do whatever we need, blood pressures, however, right now, I’m taking their word. It’s not that I don’t trust them but taking their word, are they monitoring their pressure, like things that I can’t see. I’m just taking whatever I can from them, right?”*

*[PP, KI027, AB].*

While patients understood the switch to phone visits, many patients shared that they preferred the visits in-person, as they felt they did not accomplish as much and were less comfortable to share personal information.

*“My visit happened during the COVID-19 restrictions. Preferably my visit would have been in person, but a phone visit was a reasonable alternative.”*

*[Patient, female, AB].*

*“I think this program should have a do over when COVID is over, because in person visits work better. It is easier to talk face to face as opposed to phone calls, we tend to keep a lot of things to ourselves when on a phone call.”*

*[Patient, female, NL].*

However, switching the visits from in-person to over the telephone also had advantages for patients, as they could connect with their PPs from the safety of their home, did not have to take time off work nor had the hassle of traffic and parking. PPs also found the visits more efficient and had to deal with less distractions to the prevention visits.

*“But [the prevention visits] were really good by phone. Some were quick calls, some were longer. But I know that patients appreciated it because it was more convenient for them. They didn’t have to take time off work to come down or pay for parking because we have paid parking around our building. So, I know the patients appreciated it.”*

*[PP, KI037, AB].*

Patients appreciated that the program was able to continue remotely and that they had the opportunity to talk to a healthcare professional, especially when they did not feel safe to come into the clinic or when physicians were not available for prevention and screening appointments.

*“I think the idea was to see if individual appointments would work to stay on top of screenings. My doctor was doing virtual appointments long before this study or COVID and this is a great idea for aging immunocompromised patients - do not need to present at office full of sick people.”*

*[Patient, female, ON].*

*“[The PP] is such a nice person to talk with. I always feel her full support and non-judgmental attitude. It has also been helpful to have her to ask questions to since I have not had a doctor’s appointment since the pandemic began.”*

*[Patient, female, AB].*

As a result of having to switch the prevention visits to over the telephone only, physicians, PPs and patients reported that a hybrid model would work best if BETTER WISE was to continue, especially if the first visit could take place in-person to build a trusting relationship and if PPs’ and patients’ preferences could be taken into account.

*“The disadvantages that I see is if you’re trying to establish a new relationship that might be challenging to do over the phone only. And so, in that respect, I think a more blended approach would be helpful, you know, i.e. in-person visit for the first time and then flexibility for the next few or something along those lines.”*

*[PP, KI022, AB].*

### Theme 2: lack of access to preventive care and delays of screening tests

After March 2020, all provinces participating in the BETTER WISE study (Alberta, Ontario, and Newfoundland & Labrador) were directed by their respective governments to pause non-essential services (e.g. screening and routine diagnostic testing), which had a direct impact on BETTER WISE, as the primary outcome measures included completion of screening tests (e.g., fecal immunochemical test [FIT], mammography, Pap tests) and blood work for eligible patients.

*“Some of [the patients] are overdue for their cancer screening, simply because in the pandemic, the resources were closed down for the safety and the precautions against the virus.” [PP, KI029, ON].*

*“At the very beginning things were really locked down and a lot of services and things were postponed and delayed. And this affected also as well some of the referral programs that we would have gone to. So, for example, one really popular program, the exercise program through the primary care network that we use a lot and was extremely well received by patients unfortunately was halted. You know, a lot of these different programs that we referred to were halted. And also, patients were much more apprehensive going for screening labs or going for tests and things like that. And it was quite disruptive right at the beginning.”*

*[Physician, KI047, AB].*

The reopening of non-essential services occurred at different times for each clinic, but most adopted a slow, gradual approach. Enhanced safety measures and COVID protocols reduced efficiency of patient flow through clinics and laboratory collection sites. Sites also employed waitlists and appointment-only visits to reduce walk-ins and traffic.

*“Lab work is not as bad now. It was in the beginning, but now of course it’s taking delay of course, because it’s taking a lot of time, because people can’t just show up and get a number and such to be tested. They have to call ahead, book an appointment and show up when they’re told to show up.”*

*[PP, KI028, NL].*

At the completion of this study, clinics are still in the process of catching up with the backlog of outstanding tests, as provinces are still in the midst of an ongoing pandemic and associated restrictions.

*“So, we really haven’t done a lot of screening. It’s actually – it’s on our radar right now and it’s giving some of us nurses sleepless nights of just how far behind our screening is. We’ve always been on top of our screening and it’s been the focus in the clinic. And at the moment it’s just getting by every day. So, it’s been a real – yeah. It stresses me.”*

*[PP, KI045, AB].*

Some physicians commented that those participating in BETTER WISE might be less behind than other patients, as they are more aware of their outstanding screening tests.

*“As I say, my postulate is reviewing on that Health Quality Council of Alberta (HQCA) data there will be an increasing lag in the degree to which the population is up to speed for their screening and primary prevention maneuvers. And I suspect again people who have been part of the BETTER WISE, that will be less. So, I do think that having the structure that works within the setting of practice to be able to help catch that up may be very helpful.”*

*[Physician, KI047, AB].*

Even after some clinics reopened screening tests to the general population, PPs reported that some patients did not feel comfortable coming in for routine screening tests. Both patients who were low-risk and deemed it not immediately necessary as well as those who were high-risk (e.g. immunocompromised) and did not want to expose themselves, were hesitant to complete screening.

*“When the programs, you know, were running as per their direction, our challenge was that there were patients that were not willing to come in for a Pap test, for example, or to go for a mammogram, not sort of willing to engage in the normal preventive care screening that they would had there not been a pandemic.”*

*[Physician, FG006, ON].*

*“There’s still obviously a certain group of the population that is not going. And those are the people that are very high risk. That have multiple comorbidities or who are immune compromised. So sometimes someone’s going to their house to do that test for them, if they cannot afford that service, so they’ve got to wait.”*

*[PP, KI029, ON].*

Since many patients were unable to complete their screening tests over the course of the BETTER WISE study, the outcome measure determining the percentage of completed eligible screening items will likely be lower than predicted. It is an unfortunate circumstance of the ongoing pandemic that we will not be able to determine if participating in the BETTER WISE study would lead to an increase in screening actions completed.

### Theme 3: changes in primary care providers’ availability and shift in priorities

The pandemic impacted existing clinic protocols and shifted primary care providers’ availability and priorities. This included prioritizing more acute patient concerns, some providers being redeployed to COVID-19 testing sites and vaccination clinics, and strategizing how to use office space to limit risks for patients and staff.

*“I think the way that we prioritized visits or concerns have also changed. So, from the very beginning of the pandemic, because time in the office was limited to space patients out of the office, the number of us, we were not bringing these preventative health or physical exams into the office because we needed that office time to see these patients that we deemed over telephone needed to be assessed or had more acute or symptomatic concerns. So, I think what we saw in the office and how we practice in general changed because of the pandemic.”*

*[Physician, FG005, ON].*

The focus on acute care and pandemic-related care resulted in decreased prevention and screening efforts.

*“[W]e just managed to have a global emergency in the middle of this study where the relative importance of screening and primary prevention on lower-risk people was not as much of a priority. And so, I think it’s just really unfortunate. I do think there’s going to be a lot of catch up to do.”*

*[Physician, KI047, AB].*

*“Screening of non-urgent things just went right off the radar. (…) And we couldn’t even get anyone for urgent stuff much less for screening stuff. So, it had to have had an impact on how the physicians approach those recommendations and you know, the whole screening thing would have been significantly delayed because of the COVID restrictions over the last 18 months.”*

*[Physician, KI040, NL].*

PPs also observed a change in priorities in patients, as many reported losing their jobs, having to homeschool their children, and feeling the stress of the uncertainties of the pandemic.

*“[T]here was a lot of other high priority items that families were dealing with, like kids suddenly being at home or because all the schools were closed or many Ontarians lost their jobs. So, there was just other factors, I think. And I remember calling and that happened, that was very true for my control group. I didn’t actually have people participate because they were just saying, “listen, like I just too much is going on. I don’t really have time to focus on this right now”. So, big mindset, different shifting priorities.”*

*[PP, KI034, ON].*

Particularly interesting was how the shift in priorities affected patients’ focus on their health. For example, PPs found some patients were even more health-conscious and wanted to focus on not getting sick, while other patients no longer had time to focus on their health, as they were dealing with issues related to the pandemic.

*“I think in general it’s not just the clinic staff but the patients as well and because of COVID-19 they’re thinking about their health more and are more conscious about it.”*

*[PP, KI023, AB].*

*“So, like while people were losing their jobs right, left and centre and they’d be losing their ability to getting food and financial security and mental health. So, there were some people that just gave up on their goals because of the pandemic, they were like, “It’s not worth it, I am too stressed. I can’t handle it; this pandemic is too much”. So, I think that could have affected the – like it wasn’t just that we couldn’t do the screening, but it was also just that the stress of the pandemic was affecting our patients so they might not have the mental capacity or the financial capacity or the social capacity to address these issues when there were so many other stressors because of the pandemic.” [PP, KI033, AB].*

The shift in priorities was identified when PPs checked in with patients about goal setting. Some patients could no longer work on their goals due to external circumstances (e.g., closure of gyms), some patients just felt overwhelmed by the idea of setting goals, and some found it helpful to change their goals to deal with the stress of the pandemic.

*“A lot of [the patients] just felt overwhelmed and didn’t feel like they – it was a priority at that point for them to focus on, you know, getting outside for a walk or eating more vegetables or whatever it was. So, I think a lot of the conversations about behaviour change were focused on that and kind of just maintaining where they’re at or just really focusing on those small wins – those, you know, small things that they can do to keep focusing on their health and getting them closer to reaching their goal at some point.”*

*[PP, FG007, ON].*

*“So, it impacted the type of goals people set. (…) They would have been broader goals, I think, and – but these were more focused on, like mental health goals, and because of the isolation and how to deal with that. (…) The goal setting changed, in some situations, to – more to, you know COVID–related issues.”*

*[PP, KI020, NL].*

### Theme 4: mental health impacts of the pandemic on patients and primary care providers

The fourth and last theme that emerged from the qualitative study was the impact of the COVID-19 pandemic on patients’ and primary care providers’ mental health. Prevention Practitioners perceived patients to have more mental health concerns, such as an increase in feelings of loneliness, fear, anxiety, and depression. This may have led to decreased engagement with their health and some patients withdrawing from the program.

*“I definitely think there was an increase in mental health disorders, like anxiety, depression. And I think it might have exacerbated some other types of mental health disease (…). The isolation certainly affected people, especially those living in the city that weren’t able to connect with family. There was a lot of loneliness I think, that was pretty hard to get through.”*

*[PP, KI029, ON].*

*“Some people did decide to withdraw their consent, which has been too bad. And it’s just for different types of reasons but sometimes it’s just them feeling I guess a little bit overwhelmed with the different things that were going on in their life and they felt like this was just, you know, something else that they had to do, like another – it felt more like a chore for them to be in the program than something that could maybe support them through those more difficult times and to making sure that they are staying healthy.”*

*[PP, KI030, ON].*

According to PPs, patients felt appreciative of the opportune check-ins. The visits provided connection and a chance for social interaction, which was a welcome change from the feelings of isolation that the pandemic created. This was especially reported by high-risk groups such as seniors or individuals who lived alone.

*“I think the benefits of this study certainly was it allowed me to connect to patients who I think just appreciated a phone call from someone from our office, just to make sure that they felt they were being taken care of (…) I think the older group within the study also appreciated it, because the seniors of course, many of them have been isolating and were just lonely.”*

*[PP, KI029, ON].*

*“My capacity to follow the eating and lifestyle commitments has waxed and waned through my time in this program, partly due to the effects of the pandemic, but it has been a valuable and steady support having PP check-ins, which give me a lift and inspiration to do my best with this.”*

*[Patient, female, AB].*

Primary care providers, such as the PPs and physicians, were also affected by the pandemic and reported increased stress and burnout/COVID fatigue.

*“The stress level at different times has been very much increased with different things that have gone on, the different changes, the policies, trying to keep up with things, patients trying to understand the different changes, that I’m trying to, like, explain to them. Yeah, just the whole process of it has been exhausting.”*

*[PP, KI031, ON].*

PPs and physicians identified support from the BETTER WISE team as the reason that BETTER WISE was able to be successfully completed despite the disruption of the COVID-19 pandemic. PPs who had the heaviest work load with study procedures, as well as reaching and meeting with patients, appreciated assistance and guidance from the BETTER WISE team, namely monthly check-ins to clarify questions that arose and to troubleshoot where needed around transition periods or changes in protocol.

*“Just having you guys, you know, helping us through the process when certain changes do happen such as the pandemic and adapting to that, like I think you guys adapted really well and you guys helped us adapt really well.”*

*[PP, KI023, AB].*

PPs also reported how vital support from their clinic team and the participating physicians was to how smoothly the program ran in their clinic. They cited daily COVID-19 meetings to discuss the changes in protocol as key to the operations of the clinic and valued administrative help and interdisciplinary teamwork.

*“And it was just really important to have supportive physicians and supportive managers to go through this process and a supportive team as well. Like there’s everything from people helping me scan in the consent forms to I had someone help me with phone calls at the very beginning.”*

*[PP, KI033, AB].*

*“I think our unit was always, through the whole project, was very committed to project restoration. Even when, you know, we hit some roadblocks, I think we really tried to manage them as best we could, and that – and that was because we did believe in the project and we felt that it was a benefit to our patients.”*

*[Physician, FG006, ON].*

## Discussion

The COVID-19 pandemic has been challenging for primary care and primary prevention and screening [2, 3]. In March 2020 a public health state of emergency was declared, and all non-essential services were closed. There was a shift of duties and services to COVID-19 related activities, and this had an impact on the ability of participating clinic sites to provide ongoing committed PP resources to the study. Several sites were no longer able to continue in the project due to staff deployment to COVID-19 activities. The COVID-19 imposed restrictions substantially impacted health care at the practice, provider, and patient level. Health care providers had to shift their focus to COVID-19 at a cost to prevention and screening activities. During the shut-down, non-essential services were no longer available, including resources aimed to improve health such as exercise facilities, and screening activities. In this qualitative study, we identified four themes related to how the COVID-19 pandemic impacted the BETTER WISE project, based on primary care providers’ and patients’ responses.

The first theme involved shifting from in-person visits to visits over phone, which provided an opportunity for primary care providers to stay in touch with patients and continue with the prevention visits safely. Although phone visits were not suitable for everyone, some patients thought that phone visits were a convenient option with many advantages, such as not having to take time off work, not paying parking fees, and the safety of staying home. The shift to virtual care during the COVID-19 pandemic was a phenomenon that many primary care clinics experienced in Canada [14–16]. A review of the international literature including 17 studies indicated that telehealth was one of the main strategies to maintain safe, continuous, and accessible primary care [17]. Our findings suggest that a hybrid model could work well in primary care, particularly in the context of prevention, screening and lifestyle counselling. Similarly, several studies suggested that primary care teams found virtual visits useful and planned to have a percentage of visits with patients over the phone or online as a viable future option in primary care [16, 18]. In our study, one important element we identified was the connection between primary care providers (i.e., physicians and PPs) and patients. A trusting relationship between patients and primary care providers was key amidst the uncertainties of a pandemic and has also been identified as important in public health [19], family practice [20], as well as in previous iterations of BETTER [10, 21].

The second theme, patients’ lack of access to preventative care and delays of screening tests that they were due for, is reflected in the current emerging literature that suggests that the lack of face-to-face interactions between patients and their physicians will lead to missing diagnoses of cancers and heart conditions [4], the delay of screening will contribute to patients’ disease severity [5], and increase the mortality of cancers such as colon cancers worldwide [6]. To mitigate these delays, some physicians in this study have suggested to prioritize high risk patient groups for prevention and screening. Similarly, Zheng and colleagues [5] recommend to “identify and prioritize vulnerable patients and bring them back for required procedure(s) to reduce the risk adverse outcomes related to the delay”. A structured approach such as BETTER WISE could help identify high risk patients and keep patients out of emergency rooms and hospital beds. It would also be worthwhile for future research to explore patients’ own perspectives on how the COVID-19 pandemic affected their access to and experience with primary prevention and screening for cancer and chronic diseases.

The third theme, changes in primary care providers’ availability and shift in priorities, is closely related to the previous theme of patients’ lack of access to prevention and delay of screening tests. For instance, a Pap test would have been impacted, as patients would not have been able to book an appointment with their family doctor for the testing procedure. Primary care providers had to be strategic in which patients could be seen and had to shift their focus to COVID-19 testing, vaccination, and acute care issues. Glazier and colleagues [15] from Ontario found that in comparison to 2019 (before the COVID-19 pandemic) the number of visits between patients and primary care physicians decreased by 28% overall, with most visits taking place virtually and in-person visits decreasing by 80%. A BETTER WISE visit by a PP offers the opportunity for primary care clinics to reach more patients and to catch up on outstanding prevention and screening, as well as lifestyle counselling.

Lastly, our fourth theme identified the mental health impacts of the COVID-19 pandemic on patients and primary care providers. Primary care providers observed an increase in patients’ feelings of loneliness, anxiety, and depression and a surge in addictions. This is in line with a longitudinal study in England that found a deterioration of mental health especially during lockdowns [22]. Similarly, in a study on interprofessional primary care, Donnelly et al. [14] found that the most reported health conditions were addictions concerns and mental health, totaling over 25% of all conditions. Interestingly, Donnelly and colleagues [14] observed that the increased focus on mental health and addictions meant less attention to patients’ management of their physical chronic disease, which will have long term effects. The PPs in this study reported that their phone calls were appreciated by patients and often used as opportunities to discuss strategies to mitigate stress and health concerns during the pandemic. We also found that our primary care providers, including PPs and physicians, noticed higher stress levels and commented on the risk of burnout and COVID fatigue, which has been reported by other primary care teams who had to act quickly with little support or preparation, an experience that was stressful and overwhelming [2, 3]. Support by clinic leaders, the BETTER WISE team, and good communication in interdisciplinary teams were identified as facilitators to mitigate stress and burnout. Our study findings contribute to the growing body of research, which suggests that strong teams, characterized by good communication and good relationships, are essential to avoid burnout for primary care teams in a pandemic [23].

### Strengths and Limitations.

We identified both strengths and limitations in this qualitative sub-study. This study’s strength is that we could include perspectives from patients and primary care providers from diverse clinic settings (rural, remote and urban) in three different provinces (AB, ON, and NL) in Canada. It is a possible limitation that patients were enrolled in the BETTER WISE cRCT by invitation on a voluntary basis, which may represent a selective sample of the population with potentially different interests in prevention and motivation for change than patients who received our invitation, but chose not to participate.

## Conclusion

In conclusion, the COVID-19 pandemic had a substantial impact on practices, providers, and patients. Health care providers described feeling exhausted with the stress of providing health care and the additional services and resources that were required to address the COVID-19 pandemic. Patients, particularly those that were no longer able to connect with their families, described isolation and loneliness, which had a negative impact on their mental health. COVID-19 restrictions and loss of non-essential services prevented the ability to provide cancer and chronic disease prevention and screening activities. The restrictions also impacted the BETTER WISE study by forcing visits to be delivered remotely, pausing screening tests, changing procedures, shifting priorities, and affecting the mental health of primary care providers and patients. Despite the screening restrictions imposed, and resource and mental health strains, many primary care settings participating in BETTER WISE were able to pivot to phone and virtual visits to be able to provide the best primary prevention and screening care possible to their patients. Support from multiple levels, including the BETTER WISE study team, physicians, and other clinic team members allowed the PPs to be able to continue in their role. These efforts provided patients with opportunities for connection at a time of isolation and continuity of care at a time of disruption.

By adapting to the changing climate and providing support to individuals, the BETTER WISE study was able to be completed in the summer of 2021. This sub-study is an example of one of the many research projects that was challenged by the COVID-19 pandemic. However, our results suggest that a structured primary prevention and screening program can be delivered remotely and it is possible to provide preventative care to patients with some adaptation and flexibility, which is key in an evolving medical landscape.

## Data Availability

The datasets generated or analysed during the current study are not publicly available due to concerns that participants could be identified. Patient data is available from the corresponding author on reasonable request.

## Abbreviations

AB: Alberta, Canada
BETTER: Building on existing tools to improve chronic disease prevention and screening in primary care
BETTER WISE: Building on existing tools to improve cancer and chronic disease prevention and screening in primary care for wellness of cancer survivors and patients
CCDPS: Cancer survivorship and chronic disease prevention and screening
COVID-19: Coronavirus disease 2019
cRCT: Cluster randomized controlled trial
FG: Focus group
FIT: Fecal immunochemical test (screening test for colon cancer)
KI: Key informant interview
NL: Newfoundland and Labrador, Canada
ON: Ontario, Canada
RCT: Randomized controlled trial
S.M.A.R.T.: Specific, measurable, attainable, realistic, time-based
Pap: Papanicolaou test (screening test for abnormal cells in cervix)
PP: Prevention Practitioner
